# Do Black Women’s Religious Beliefs About Body Image Influence Their Confidence in Their Ability to Lose Weight?

**DOI:** 10.5888/pcd14.170153

**Published:** 2017-10-19

**Authors:** Alexandria G. Bauer, Jannette Berkley-Patton, Carole Bowe-Thompson, Therese Ruhland-Petty, Marcie Berman, Sheila Lister, Kelsey Christensen

**Affiliations:** 1 University of Missouri-Kansas City, School of Medicine, Kansas City, Missouri; 2 University of Missouri-Kansas City, Department of Psychology, Kansas City, Missouri

## Abstract

**Introduction:**

Black women are disproportionately burdened by obesity but maintain body satisfaction and strong religious commitment. Although faith-based weight-loss interventions have been effective at promoting weight loss among blacks, little is known about how body image and religious views contribute to weight-related beliefs among religious black women. The purpose of this study was to examine whether demographic and health history factors, religious involvement, and beliefs about body image could explain motivation and confidence to lose weight among a church-affiliated sample of black women.

**Methods:**

We recruited 240 church-affiliated black women aged 18 to 80 years (average age, 55 y; SD, 12.3) in 2014 from 6 black churches that participated in a larger study, Project FIT (Faith Influencing Transformation), a clustered, diabetes/heart disease/stroke intervention among black women and men. We used baseline data from Project FIT to conduct a cross-sectional study consisting of a survey. Variables approaching significance in preliminary correlation and χ^2^ analyses were included in 2 multiple linear regression models examining motivation and confidence in ability to lose weight.

**Results:**

In final regression models, body mass index was associated with motivation to lose weight (β = 0.283, *P* < .001), and beliefs about body image in relation to God predicted confidence to lose weight (β = 0.180, *P* = .01).

**Conclusion:**

Faith-based, weight-loss interventions targeting black women should emphasize physical well-being and highlight the health benefits of weight management rather than the benefits of altering physical appearance and should promote positive beliefs about body image, particularly relating to God.

## Introduction

Black women are disproportionately burdened with obesity. An estimated 80% are overweight or obese (body mass index [BMI] ≥25) (1,2), and cultural beliefs may contribute to high rates of obesity among black women (3,4). Black women’s beliefs regarding an ideal body shape (ie, being shapely and curvy; having large breasts, hips, and buttocks) are perceived to be more attractive in the black community but tend to differ from those of mainstream archetypes (3).

Many studies report that black women are less concerned about weight than women in other racial/ethnic groups (4), that they prefer large body shapes (5), that they have high levels of body satisfaction and self-esteem while overweight or obese, and that they tend not to believe that losing weight will improve quality of life (4,6–9). Furthermore, studies showed that black women who lost weight were concerned that they appeared too thin or unwell (10) and worried that a petite body frame might be viewed negatively (eg, as scrawny) (3). Conversely, some studies found that overweight and obese black women have weight-related concerns and would like to lose weight (11,12), particularly because of the effect of their weight on their health (13). In one study, obese black women reported that a larger body size could be healthy and attractive but expressed interest in losing weight and were self-conscious about their body size (14). Still, cultural norms that promote body acceptance, inaccurate perceptions of body size, and limited knowledge of weight-related health problems (12) may reduce motivation and confidence to lose weight. Little is known about what influences motivation and confidence to lose weight among black women, especially in settings that could extend the reach and effect of black female-focused, culturally tailored, weight-loss interventions.

Black churches have the potential to shift cultural body image norms and provide support for weight loss among black women. In national studies, more than 80% of black women described religion as very important, and almost 60% reported attending church services weekly (15). Most black churches have health or outreach ministries (eg, food and clothing pantries, day care) that can be tapped by health promotion programs to reach women in the communities they serve (16–18). Additionally, most churches promote the scripture that the body is made in the image of God and is the temple of God, encouraging members to take care of their bodies (19).

Despite the growing number of obesity studies in black churches (17), virtually no studies have examined potential cultural and health contributors to weight-related beliefs among black women church-goers. Improving understanding of these beliefs could inform the design of tailored weight-loss interventions. The objective of this study was to explore whether demographic and health history factors, religious involvement, and body image beliefs would predict motivation and confidence to lose weight in a church-affiliated sample of black women.

## Methods

We used a cross-sectional design to examine baseline data that were collected over a 6-week period in October and November 2014 as part of larger intervention, Project FIT (Faith Influencing Transformation), a religiously tailored intervention for diabetes/heart disease/stroke education, screening, and linkage to care conducted in urban areas of Kansas City, Missouri, among black women and men. Analyses for Project FIT were performed in November 2016 (results have not been published). Participants for our study were recruited from 6 black churches that participated in Project FIT through announcements from pastors and other church leaders during church services and through church outreach events (eg, food pantry).

We recruited 240 black women from Project FIT. The response rate was 94% across all predictor variables and 98% for outcome variables. Participants met the following eligibility criteria: self-identified as black and female, were aged 18 to 80 years, did not have sickle cell anemia, were not pregnant or planning to become pregnant, and were willing to participate in a survey. Surveys were paper and pencil and assessed health-related beliefs and behaviors. Surveys took approximately 30 to 45 minutes to complete, and participants received $20 in cash for completing the survey. Participants provided written informed consent, and the University of Missouri–Kansas City institutional review board approved the study.

### Survey measures

Participants were asked to provide information on age, education level, income, marital status, and whether they had children. Income and marital status were dummy-coded to compare married women with all other groups (eg, single, widowed, divorced women) and to compare women with a monthly household income of more than $3,000 to women with a household income of $3,000 or less. Having children was measured as a dichotomous (yes/no) variable.

Health history variables collected were health insurance coverage, body mass index (BMI; kg/m^2^), health care visits, diagnosed health conditions, and perceived stress. To calculate BMI, a member of the research team used a SECA stadiometer and scale (SECA) to measure height and weight without shoes.

Three survey questions developed for this study assessed health care visits over the past 12 months (ie, physician visits, emergency department visits, and hospitalizations; Cronbach α = 0.46) with responses ranging from none, once, twice, or 3 or more. Responses were summed, with total scores ranging from 0 (no health care visits) to 12 (nine or more health care visits). Fifteen dichotomous (yes/no) questions assessed diagnosed health conditions (eg, high blood pressure, diabetes, depression; Cronbach α = 0.50). Responses were summed, with total scores ranging from 0 (no diagnoses) to 15 (maximum number of conditions). Perceived stress was measured by using questions adapted from the Perceived Stress Scale, which was designed for ease of use with community-member participants (Cronbach α = 0.84–0.86 in previous studies) (20). Fourteen questions asked participants to rate their experiences with stress (eg, “Felt that you were unable to control the important things in your life”; 1 = never to 5 = very often; Cronbach α = 0.95). Questions with positive answers (eg, “Dealt successfully with irritating life hassles”) were reverse-scored. Summed scores ranged from 14 (low stress) to 70 (high stress).

To assess religiosity, one question asked how many years participants had been church members. Seven questions, adapted from the Religious Background and Behavior Scale (21) and used in previous studies among faith-based black populations (Cronbach α = 0.77) (22), assessed participants’ religiosity (Cronbach α = 0.74 for this study). The first question asked participants to describe their religious identity (ie, 1 = atheist, 2 = agnostic, 3 = unsure, 4 = spiritual, 5 = religious). Six additional questions asked participants about frequency of religious behaviors (eg, thought of God, prayed, attended a worship service) and were ranked on a scale of 0 (never) to 8 (more than once a day). Summed scores for the 7 religious identity and behavior items ranged from 7 (low religiosity) to 53 (high religiosity).

Participants were asked to rate satisfaction with their overall appearance and with their arms, waist, thighs, and buttocks on a scale of 1 (highly not satisfied) to 7 (highly satisfied) (Cronbach α = 0.92 for this study). Responses were summed, so that total scores ranged from 5 (low body satisfaction) to 35 (high body satisfaction).

Five questions assessed participants’ beliefs about their body in relation to God (eg, belief that “My body is a temple of God” and that “My body is a gift from God.”). Responses were rated on a scale of 1 (strongly disagree) to 7 (strongly agree) (Cronbach α = 0.74 for this study). Responses were summed, and scores ranged from 5 (no body image beliefs in relation to God) to 35 (strong body image beliefs in relation to God).

Two questions ranked how motivated participants were to lose weight and how confident they were that they could do so, both on a scale of 0 (not at all confident or motivated) to 10 (very confident or motivated).

### Data analysis

Descriptive statistics were used to examine all variables. Preliminary analyses (ie, correlation, χ^2^) were performed to examine associations between predictor variables (ie, demographics, health history, religious involvement, body image beliefs) and motivation and confidence to lose weight. We used SPSS version 22.0 (IBM Corp) to enter variables that were significantly associated or approached significance (ie, *P* ≤ .10) with motivation or confidence to lose weight into 2 separate multiple linear regression models with the first model examining motivation to lose weight and the second model examining confidence to lose weight. Significance levels were set a priori for both regression models at *P* < .05. Categorical variables (eg, marital status, income) were dummy-coded. All variables included in each of the linear regression models were entered in a single step. For the purposes of this cross-sectional study, explanatory variables are referred to as predictor variables with motivation and confidence to lose weight as outcomes (23).

## Results

Participants’ average age was 55 years (standard deviation [SD], 12.3; range, 18–80 y). Most participants reported being married and having children, a college degree or higher, health insurance, and a monthly household income less than $3,000 (Table 1). Average motivation to lose weight was 7.8 on a scale of 0 to 10 (SD, 2.7) and average confidence in ability to lose weight was slightly higher (mean, 8.0; SD, 2.5; scale 0–10). Age and having children were not related to motivation to lose weight (Table 2) or confidence in ability to lose weight (Table 3). Income and marital status were both positively related to motivation to lose weight in preliminary analyses. Income also was positively related to confidence in ability to lose weight in preliminary analyses; however, neither income nor marital status predicted motivation or confidence in ability to lose weight in final regression models.

**Table 1:** Demographic and Psychosocial Characteristics of a Sample of Church-Affiliated Black Women (N = 240) in Kansas City, Missouri, 2014

Characteristic	No. (%)^a^
**Age, mean (SD), y**	55 (12.3)
**Education **	
11th grade or below	10 (4)
High school diploma or general equivalency degree	29 (12)
Some college or post-high school technical training	5 (2)
Associates degree or technical school certificate	71 (30)
College degree or higher	125 (40)
**Monthly household income, $ **	
0–1,000	24 (10)
1,001–2,000	36 (15)
2,001–3,000	55 (23)
>3,000	76 (32)
Don’t know	11 (5)
Refused to answer	14 (6)
**Marital status **	
Single, never married	60 (25)
Living with partner, not married	3 (1)
Married	83 (35)
Separated	13 (5)
Divorced	59 (25)
Widowed	22 (9)
**Children**	
Yes	201 (85)
No	37 (15)
**Years as a church member**	
0 to <2	5 (3)
2 to <5	21 (14)
5 to <10	22 (14)
10 to <20	31 (20)
≥20	74 (49)
**Health insurance coverage^b^**	
Medicare	60 (25)
Medicaid	19 (8)
Private insurance	129 (54)
Other insurance	28 (12)
No insurance	32 (13)
**Body mass index (kg/m^2^)**	
Underweight (<18.5)	5 (2)
Normal weight (18.5–24.9)	24 (10)
Overweight (25.0–29.9)	56 (24)
Obese class I (30.0–34.9)	56 (24)
Obese class II (35.0–39.9)	43 (19)
Obese class III (≥40.0)	48 (21)
**Number of health care visits in past 12 months**	
1	0
2	0
3	23 (10)
4	25 (10)
5	29 (12)
≥6	92 (38)
**Number of diagnosed health conditions**	
0	53 (22)
1	63 (26)
2	53 (22)
3	41 (17)
4	16 (7)
5	10 (4)
≥6	4 (2)

Abbreviations: —, not assessed; SD, standard deviation.

a Unless otherwise indicated, values are numbers (percentages). Percentages may total less than 100 because of rounding or missing responses.

b Percentages total more than 100 because categories were not mutually exclusive.

**Table 2:** Preliminary Associations and Linear Regression for Motivation to Lose Weight Among Church-Affiliated Black Women in Kansas City, Missouri, 2014

Variable	Preliminary Analyses			Linear Regression		
	*r*	χ^2^	*P*	β (95% CI)	SE	*P*
**Demographic characteristic**						
Age	0.052	^—a^	.43	^—b^	^—b^	^—b^
Income	^—a^	75.7	.08	−0.003 (−0.824 to 0.792)	0.410	.97
Marital status	^—a^	67.9	.046	0.133 (−0.044 to 1.54)	0.401	.06
Children	^—a^	14.1	.83	^—b^	^—b^	^—b^
**Health history**						
Body mass index (kg/m^2^)	0.218	^—a^	<.001	0.283 (0.046 to 0.135)	0.022	<.001
Health care visits	−0.018	^—a^	.82	^—b^	^—b^	^—b^
Diagnosed health conditions	−0.012	^—a^	.86	^—b^	^—b^	^—b^
Perceived stress	−0.127	^—a^	.07	−0.117 (−0.099 to 0.008)	0.027	.10
**Religious involvement**						
Years as church member	−0.076	^—a^	.25	^—b^	^—b^	^—b^
Religiosity^c^	0.037	^—a^	.59	^—b^	^—b^	^—b^
**Body image**						
Body satisfaction	−0.077	^—a^	.28	^—b^	^—b^	^—b^
Body in relation to God	0.111	^—a^	.09	0.049 (−0.038 to 0.081)	0.030	.48

Abbreviations: CI, confidence interval; SE, standard error.

a Preliminary associations were either correlation (continuous variable) or χ^2^ (categorical variable), as appropriate.

b Excluded from regression because of nonsignificant preliminary associations.

c Religious attitudes, beliefs, and behaviors.

**Table 3:** Preliminary Associations and Linear Regression for Confidence to Lose Weight Among Church-Affiliated Black Women in Kansas City, Missouri, 2014

Variable	Preliminary Analyses			Linear Regression		
	*r*	χ^2^	*P*	β (95% CI)	SE	*P*
**Demographic characteristic**						
Age	−0.003	^—a^	.96	^—b^	^—b^	^—b^
Income	^—a^	78.7	.02	0.068 (−0.320 to 1.045)	0.346	.30
Marital status	^—a^	47.8	.36	^—b^	^—b^	^—b^
Children	^—a^	6.3	>.99	^—b^	^—b^	^—b^
**Health history**						
Body mass index	0.095	^—a^	.15	^—b^	^—b^	^—b^
Health care visits	−0.114	^—a^	.14	^—b^	^—b^	^—b^
Diagnosed health conditions	−0.085	^—a^	.20	^—b^	^—b^	^—b^
Perceived stress	−.029	^—a^	.66	^—b^	^—b^	^—b^
**Religious involvement**						
Years as a church member	−0.029	^—a^	.66	^—b^	^—b^	^—b^
Religiosity^c^	0.090	^—a^	.20	^—b^	^—b^	^—b^
**Body image**						
Body satisfaction	0.097	^—a^	.17	^—b^	^—b^	^—b^
Body in relation to God	0.179	^—a^	.01	0.180 (0.019 to 0.113)	0.024	.01

Abbreviations: CI, confidence interval; SE, standard error.

a Preliminary associations were either correlation (continuous variable) or χ^2^ (categorical variable), as appropriate.

b Excluded from regression because of nonsignificant preliminary associations.

c Religious attitudes, beliefs, and behaviors.

Participants’ average BMI was 32.8 (SD, 8.5; range, 18–54). In preliminary analyses BMI was significantly associated with motivation but not with confidence. BMI significantly predicted motivation to lose weight in the regression model.

Most participants (87%) had visited their physician for a regular checkup in the past year, and 44% had visited their physician 3 or more times (overall scores for health care visits ranged from 3 to 10, scale 0–12). The most common place for routine medical care was a physician’s office or health maintenance organization (73%). More than half (52%) had visited an emergency department, and 39% had been hospitalized in the past year.

Participants reported an average of 1 or 2 diagnosed health conditions (mean, 1.7; SD, 1.5; scale 1–15; range, 0–6 conditions), most commonly high blood pressure (57%), high cholesterol (39%), or diabetes (24%). Diagnosed health conditions and health care visits were not associated with motivation or confidence in preliminary analyses.

Participants reported an average perceived stress score of 36.7 (SD, 7.0; range, 20–55 on the Perceived Stress Scale). The association between perceived stress and motivation to lose weight approached significance in preliminary analyses, but stress did not predict motivation to lose weight in the regression model. Perceived stress was not associated with confidence in ability to lose weight in preliminary analyses.

The average length of time the woman had been a church member was 23 years (SD, 18.6; range, 1–72 y). The average religiosity score was 46.4 *(*SD, 5.7; range, 21–53; scale 7–53). Most participants described themselves as religious (84%) and attended church at least weekly (93%). More than once per day, many participants thought of God (85%), prayed (75%), meditated (43%), or had direct experiences with God (43%); 38% read scriptures or holy writings almost daily. Religiosity and years as a church member were not significantly associated with motivation or confidence in ability to lose weight in preliminary analyses.

Average combined body satisfaction was 21.8 (SD, 7.9; range, 5–35; scale 5–35). Body satisfaction was not associated with motivation or confidence to lose weight in preliminary analyses.

Participants had very strong body image beliefs about their bodies in relation to God (mean, 32.4; SD, 6.8; range, 5–35, scale 5–35). In preliminary analyses, body image beliefs in relation to God approached significance for motivation to lose weight and were significantly associated with confidence to lose weight. In the regression model, body image beliefs in relation to God predicted confidence to lose weight.

## Discussion

To our knowledge, this study is among the first to examine body image and religiosity, demographics, and health history as predictors of motivation and confidence in ability to lose weight among church-affiliated black women. We found that women in this study were highly motivated and confident in their ability to lose weight. Moreover, BMI significantly predicted motivation to lose weight, which was an important finding considering the high levels of overweight and obesity among this study’s church-affiliated participants and among similar populations in other church-based studies (16,17,24).

Slightly more than one-fourth of participants had been diagnosed with at least one health condition, most commonly high blood pressure, high cholesterol, or diabetes, which is consistent with previous estimates (1). Although overall poor health has been shown to motivate weight loss among black people (10,11,13), diagnosed health conditions among black women in our sample were not related to motivation or confidence in ability to lose weight. Previous research indicated that concerns about developing a chronic illness drove interest in weight-loss among black women (11,13) and that preventing or managing chronic illnesses was a primary reason to lose weight (10). Still, other studies showed that people living with chronic diseases tend to have little interest in weight loss (25), particularly because chronic illnesses can result in financial problems, pain, fatigue, limited physical functioning, or depression (26), which can inhibit weight loss. Moreover, most participants visited their physician for a regular checkup in the past year, and nearly half saw their physician multiple times. Slightly more than half of participants had visited an emergency department, and more than one-third had been hospitalized in the past year. Given the growing focus on patient-centered medicine and the high rates of contact with health care services among women in our sample, consideration should be given to how culturally tailored messages can be incorporated into physician visits or hospitalizations to increase motivation and confidence in ability to lose weight among black women at risk for, or living with, chronic diseases. Additionally, opportunities may exist for pastors and other church leaders to encourage weight loss when making hospital visits with their members.

Perceived stress did not predict motivation or confidence in ability to lose weight. Previous studies of black women demonstrated a direct relationship between stress and consumption of larger portions of unhealthy foods (13). It is likely that the perception of stress is related to behaviors that can immediately mitigate its effects (eg, emotional eating) (27) and may dampen resolve to lose weight. Black women are also particularly susceptible to stressors (eg, racism, discrimination, low pay) that can exacerbate weight gain (28). Future studies should continue to explore the influence of perceived stress on weight management attitudes and behaviors.

Participants were highly religious, and consistent with other church-based studies, they attended church frequently (15,18). However, religiosity was not associated with motivation or confidence in ability to lose weight, which is likely due to a ceiling effect (ie, very high religiosity across the sample). Similarly, time as a church member was not associated with motivation or confidence in ability to lose weight. It has been suggested that the black church environment can influence weight-related health beliefs and behaviors, including exerting pressure to consume large portions or unhealthy foods at church events (24). Recommendations for weight-loss interventions designed for black people include faith-based changes to the church culture and environment (17,19), including incorporation of church policies to limit high-fat, high-calorie, and sodium-rich foods at church events. Faith-based programs may also benefit from efforts to reshape the health-related climate in church settings by increasing social support for healthy behavior change and promoting acceptability of weight-loss behaviors.

Despite the high levels of overweight and obesity in our study sample, participants reported moderate levels of body satisfaction, which is consistent with previous studies that found positive body image perceptions among obese black women (6,7,9,). Yet, body satisfaction was not related to motivation or confidence in ability to lose weight. This finding is somewhat inconsistent with a previous study of overweight and obese women from a diverse sample that found that body satisfaction was the strongest predictor of current weight-loss efforts (29). Also, participants demonstrated equal body satisfaction across different body areas, which contrasts with the literature that suggests black women are not consistently satisfied with their bodies across all areas (11). These conflicting findings suggest that much remains to be learned about body image and motivation and confidence in ability to lose weight among church-affiliated black women.

Participants had strong beliefs about body image in relation to God, which were associated with motivation and confidence and significantly predicted confidence in ability to lose weight. Qualitative studies of church-affiliated black people have shown that believing that one’s body is a temple of God was related to engagement in healthy behaviors (eg, abstaining from alcohol, tobacco, and drugs) (19). Faith-based weight-loss interventions for black women could be tailored to promote positive, faith-based attitudes about the body while encouraging preventive health behaviors (eg, physical activity, healthy eating).

This study had limitations. It was not guided by hypothesis or theory. Instead, we took an exploratory, stepwise approach for the inclusion of variables to be analyzed. Additionally, this study was cross-sectional, which limits causal inferences. Still, this study had several strengths, including a comprehensive list of demographic, health history, religious, and body image variables of a diverse church-affiliated sample of black women with representation across age, income, and marital status. This study also contributes to the literature on motivation and confidence in ability to lose weight among church-affiliated black women, a population with high rates of overweight and obesity. Recognition of the influence of BMI and body image beliefs in relation to God on motivation and confidence in ability to lose weight presents opportunities for black faith communities to tap this inherent cultural aspect of religiosity and church tenets to reduce the burden of obesity among black church-affiliated black women.
